# Femtosecond laser-assisted cataract surgery versus conventional phacoemulsification surgery: visual outcomes with presbyopia-correcting intraocular lens

**DOI:** 10.3389/fmed.2026.1819853

**Published:** 2026-05-07

**Authors:** Chi Xiao, Runhua Peng, Zhenyang Zheng, Haiyan He, Yan Li, Zhanchi Hu

**Affiliations:** Department of Refractive Surgery, Dongguan Guangming Ophthalmic Hospital, Dongguan, Guangdong, China

**Keywords:** cataract surgery, femtosecond laser, intraocular lens implantation, phacoemulsification, presbyopia-correcting

## Abstract

**Purpose:**

To compare the visual outcomes between femtosecond laser-assisted cataract surgery (FLACS) and conventional phacoemulsification surgery (CPS) combined with presbyopia-correcting intraocular lens (IOL) implantation.

**Methods:**

A retrospective cohort study was conducted. Patients who underwent cataract surgery with presbyopia-correcting IOL implantation at Dongguan Guangming Ophthalmic Hospital between April 2023 and December 2025 were enrolled and divided into FLACS and CPS groups. Follow-up examinations were performed at 1 day and 1 month postoperatively. Uncorrected and corrected distance, intermediate, and near visual acuity (VA), spherical equivalent (SE), defocus curve, high-order aberrations (HOAs), spherical aberration, coma, trefoil, modulation transfer function (MTF), and Strehl ratio (SR) were evaluated and compared between the two groups. All outcomes were reported in accordance with international standards for reporting cataract surgery outcomes.

**Results:**

This study included 134 eyes from 134 patients, with 67 patients assigned to each group. The mean UDVA was 0.26 in the FLACS group and 0.33 in CPS group at 1 day postoperatively (*p* = 0.049). At 1 month postoperatively, all patients achieved favorable uncorrected and corrected VA at distance, intermediate, and near, alongside a significant improvement in overall visual quality compared to preoperative values (*p* < 0.001). The FLACS group demonstrated a postoperative uncorrected distance VA of 0.08 ± 0.07 logMAR, compared to 0.07 ± 0.08 logMAR in the CPS group (*p* > 0.05). The FLACS group exhibited better performance of functional VA at intermediate distance. The defocus curves of the FLACS group showed better and more stable full-range vision. Areas under the uncorrected defocus curve of FLACS and CPS groups were 1.26 and 1.18, respectively.

**Conclusion:**

Both FLACS and CPS can effectively improve VA and significantly enhance visual quality after presbyopia-correcting IOL implantation. Compared to CPS, FLACS exhibited enhanced full-range vision, more stable intermediate and near vision, as well as certain advantages in early recovery of VA.

## 1 Introduction

Cataract is one of the leading causes of visual impairment globally (1). Conventional phacoemulsification surgery (CPS) has long been the standard surgical approach for cataracts. The efficacy of cataract surgery varies among different countries and surgeons (2). One reason is that inappropriate surgical procedures increase the risk of intraoperative and postoperative complications, thereby affecting surgical outcomes. Since the femtosecond laser-assisted cataract surgery (FLACS) was introduced in 2009 initially (3), femtosecond laser technology has been widely adopted in corneal incision creation, capsulorhexis, and lens fragmentation, replacing some manually performed critical steps in CPS (4, 5).

Multiple studies have compared the safety and efficacy between FLACS and CPS, indicating comparable visual outcomes of both surgical techniques (6–10), while FLACS might be less cost-effectiveness (8). Nevertheless, FLACS demonstrated its superiority in preventing endothelial damage and controlling phacoemulsification energy to CPS in complicated cataract condition, such as hard nuclear cataract, high myopia, and vitrectomized eyes (11–13). Additionally, FLACS may be beneficial for intraocular lens (IOL) stabilization (less tilt and decentration) in the short term (14).

With the increasing demands for functional vision and satisfying visual quality at different visual distance, the designs of IOL have also been evolved from monofocal lenses to presbyopia-correcting lenses, such as multifocal and extended depth-of-focus (EDOF) IOLs. These advanced IOLs have shown promising results regarding continuous range of vision (15–18). FLACS presented lower internal aberrations and higher patient satisfaction than CPS with a diffractive multifocal intraocular lens (19). In contrast, there was no significant difference in visual outcomes between FLACS and CPS when implanting with an EDOF IOL (20). However, as an important aspect for IOL performance evaluation, defocus curve was not included in these studies when comparing.

Synergy IOL is presbyopia-correcting IOL mixed with diffractive multifocal profile and EDOF design. It achieves a full-range vision compared to trifocal IOLs, with an even better night vision (21–23). The objective of this study is to investigate whether FLACS offers advantages over CPS in terms of postoperative vision and refraction, defocus curve, and visual quality, in cataract patients implanted this presbyopia-correcting IOL.

## 2 Participants and methods

### 2.1 Participant enrollment

This retrospective, comparative study included patients who underwent cataract surgery with implantation of a presbyopia-correcting intraocular lens at Dongguan Guangming Ophthalmic Hospital between April 2023 and December 2025. All patients were diagnosed with age-related cataract and met the indications for cataract surgery. According to individual condition of patients, the presbyopia-correcting IOL (Tecnis Synergy ZFR00V) and surgical approach were recommended by the surgeon and were agreed by patients. Participants were divided into the FLACS group and the CPS group according to the surgical procedure. The inclusion criteria were as follows: (1) preoperative corneal astigmatism < 0.75 diopter (D); (2) age ≥ 45 years; (3) target postoperative spherical equivalent (SE) refraction ≤ 0.5 D. The exclusion criteria were as follows: (1) history of corneal disease, glaucoma, uveitis, and fundus diseases, or any other ocular or systemic diseases potentially affecting postoperative vision; (2) poor intraoperative cooperation, or any additional surgical procedure, such as corneal relaxing incisions and capsular tension ring implantation; (3) any monovision design or surgical design for specific visual needs. This study adhered to the principles of the Declaration of Helsinki, and the study protocol was approved by the Ethics Committee of Dongguan Guangming Ophthalmic Hospital. All participants provided written informed consent prior to surgery.

### 2.2 Cataract grading

We introduced Lens Opacity Classification System (LOCS) III for cataract grading (24). For each participant, decimal scores for cortical opacity (C), posterior subcapsular opacity (P), nuclear color (NC), and nuclear opacity (NO) were assigned by comparing corresponding colored slit-lamp and retroillumination images to standard reference images. The scales for C and P range from 0.1 to 5.9, while those for NC and NO range from 0.1 to 6.9, both in 0.1-unit increments.

### 2.3 Intraocular lens

The presbyopia-correcting IOL (Tecnis Synergy ZFR00V, Johnson & Johnson Vision, Santa Ana, California, USA) is a soft, foldable hydrophobic acrylic biconvex IOL with ultraviolet and violet light absorber and C-loop shape. The overall diameter is 13.0 mm and the optic diameter is 6.0 mm, with a center thickness of 0.7 mm (measured at 20 D). The aspheric anterior surface is wavefront-designed and achromatically optimized for enhanced contrast sensitivity. The posterior surface composes of 15 rings of diffractive echelette for extended vision. The squared edge of the IOL is frosted to promote rotation stability. The refractive index is 1.47 at 35 °C.

### 2.4 Surgical procedure

All patients underwent detailed medical history query and comprehensive preoperative examinations, including slit-lamp examination, non-contacted tonometery, fundus photography, optical coherence tomography, ocular B-scan ultrasound, IOL power measurement, and corneal topography. The Barrett Universal II formula was used for IOL power calculation with a target refraction of plano. All patients received preoperative topical 0.5% levofloxacin eye drops administered four times daily for 3 days. Thirty minutes before surgery, compound tropicamide eye drops were instilled every 10 min for a total of three doses to achieve adequate mydriasis. All surgeries were performed by the same experienced surgeon (CX). After sufficient topical anesthesia was achieved with three instillations of proparacaine hydrochloride eye drops, the sugery was initiated.

In the FLACS group, after standard disinfection and draping, the patient interface and contact lens were mounted on the Alcon LenSx femtosecond laser system. The interface position was adjusted, ocular alignment was confirmed, and the system was locked with application of negative pressure. Parameters including anterior and posterior capsulotomy marks, scan depth, and corneal incision position and depth were set and verified. After confirmation, femtosecond laser was activated to perform capsulorhexis, lens fragmentation, and creation of corneal incisions. The patient was then transferred to another operating room to perform phacoemulsification. The subsequent surgical steps were identical to those in the CPS group.

In the CPS group, after routine disinfection and draping, a main incision and a side-port incision were created. Continuous curvilinear capsulorhexis was performed, followed by hydrodissection and hydrodelineation. Phacoemulsification and cortical aspiration were carried out using the Alcon Centurion phacoemulsification system. Tecnis Synergy ZFR00V was implanted into the capsular bag. The ophthalmic viscous material was thoroughly removed, and the IOL was confirmed to be well-centered without tilt or deviation. The incisions were hydrated to ensure sealing, and intracameral injection of cefuroxime (1 mg/0.1 mL) were added. At the end of the surgery, tobramycin-dexamethasone ophthalmic ointment was applied.

Postoperatively, both groups received topical 0.5% levofloxacin, 0.3% sodium hyaluronate, and pranoprofen eye drops four times daily for 4 weeks. Tobramycin-dexamethasone eye drops were administered four times daily for 2 weeks.

### 2.5 Postoperative examinations

Cumulative dissipated energy (CDE) was recorded promptly after surgery. Uncorrected distance visual acuity (UDVA) was measured 1 day postoperatively. At one-month follow-up visit, monocular visual acuity (VA) was measured at far (5 m), intermediate (80 cm and 60 cm), and near (40 cm) distance using Snellen charts under a background luminance of 80 cd/m^2^. Manifested refraction was conducted, and VA at different distance with or without correction were recorded. Distance-corrected and uncorrected monocular defocus curve (DC) were evaluated from +1.50 D to −4.00 D with a stride of 0.50 D using phoropters. Additionally, visual quality parameters, such as total ocular higher-order aberrations (HOAs), spherical aberration, coma, trefoil, the average height of modulation transfer function (MTF), and Strehl ratio (SR), were evaluated measured using an iTrace visual function analyzer with a pupil diameter of 3 mm.

### 2.6 Statistical analysis

The raw clinical data was stored and managed using Microsoft Excel (version 2,411, Microsoft Office 2019), and statistical analysis were performed using IBM SPSS Statistics (version 26.0, IBM Corporation, Chicago, Illinois, USA). The sample size was calculated using G*Power (version 3.1.9.7) according to the HOAs results in a prospective cohort study with EDOF IOLs (20), with an effect size of 0.39, a significance level of 0.05, and a statistical power of 0.80. The standard graphs for presbyopia-correcting IOLs were drafted using GraphPad Prism (version 9.0, GraphPad Software, Boston, MA, USA). All VA records were converted to the logarithm of the minimum angle of resolution (logMAR) for mathematic calculation. Baseline characteristics were described using as mean ± standard deviation (SD) and range (minimum, maximum), while the postoperative parameters were presented as mean ± SD. The normality of continuous data was assessed using the Shapiro–Wilk test and Quantile-Quantile plot. According to the normality, the Student’s *t*-test and Wilcoxon–Mann–Whitney U test was used for comparison between the two groups, while Wilcoxon signed-rank test was utilized for intra-group comparisons. Categorical data were summarized as *n* (%) and compared between groups using the Pearson’s Chi-square test. A *p*-value < 0.05 was considered statistically significant.

## 3 Results

A total of 134 eyes from 134 patients (67 patients for each group) were included in this study. Among them, 62 patients were male and 72 were female, with ages ranging from 45 to 83 years. Table 1 summarized the baseline demographic and ocular characteristics. No statistically significant differences were observed between the FLACS and CPS groups regarding demographic information, preoperative UDVA, intraocular pressure, axial length, or implanted IOL power (all *p* > 0.05). On postoperative day one, elevated postoperative intraocular pressure (IOP) was present in 12 patients from each group. No other complications were identified during the follow-up period. As shown in Table 2, the cataract grading scores and mean CDE also indicated no significant difference between the two groups (all *p* > 0.05).

**Table 1:** Baseline characteristics of FLACS and CPS groups.

Characteristics	FLACS (*n* = 67)		CPS (*n* = 67)		*p*
	Mean ± SD	Range	Mean ± SD	Range	
Age (years)	66.4 ± 7.1	50, 80	65.5 ± 9.1	45, 83	0.520^a^
Gender (male/female)	31/36 (46/54)		41/26 (61/39)		0.083^b^
Eye (Right/left)	30/37 (45/55)		33/34 (49/51)		0.604^b^
UDVA (logMAR)	0.84 ± 0.62	0.22, 3.00	0.94 ± 0.77	0.00, 4.00	0.461^c^
IOP (mmHg)	14.4 ± 2.8	10, 23.7	14.2 ± 2.2	10, 19	0.914^c^
AL (mm)	23.89 ± 1.41	21.49, 29.26	23.78 ± 1.19	22.15, 27.36	0.660^c^
IOL power (D)	19.8 ± 3.5	7.5, 26.5	20.3 ± 3.3	7.5, 25.0	0.163^c^

FLACS, femtosecond laser-assisted cataract surgery; CPS, conventional phacoemulsification surgery; SD, standard deviation; UDVA, uncorrected distance visual acuity; logMAR, logarithm of the minimum angle of resolution; IOP, intraocular pressure; AL, axial length; IOL, intraocular lens; D, diopter.

a The Student’s *t*-test.

b Pearson’s Chi-square test.

c Wilcoxon–Mann–Whitney U test.

**Table 2:** Cataract grading scores and cumulative dissipated energy.

Characteristics	FLACS (*n* = 67)		CPS (*n* = 67)		*p*
	Mean ± SD	Range	Mean ± SD	Range	
LOCS III (C)	2.8 ± 1.2	0.5, 5.8	2.9 ± 1.3	1.0, 5.9	0.839^a^
LOCS III (NO/NC)	2.5 ± 0.9	0.1, 4.8	2.5 ± 0.9	1.0, 5.5	0.561^a^
LOCS III (P)	1.9 ± 1.2	0.1, 5.9	2.1 ± 1.1	0.5, 5.0	0.095^a^
CDE	5.62 ± 2.99	0.51, 15.71	5.87 ± 4.29	0, 26.04	0.778^a^

FLACS, femtosecond laser-assisted cataract surgery; CPS, conventional phacoemulsification surgery; SD, standard deviation; LOCS III, Lens Opacity Classification System III; C, cortical opacity; NO, nuclear opacity; NC, nuclear color; P, posterior subcapsular opacity; CDE, cumulative dissipated energy.

a Wilcoxon–Mann–Whitney U test.

### 3.1 Postoperative visual acuity

**Figure 1:**
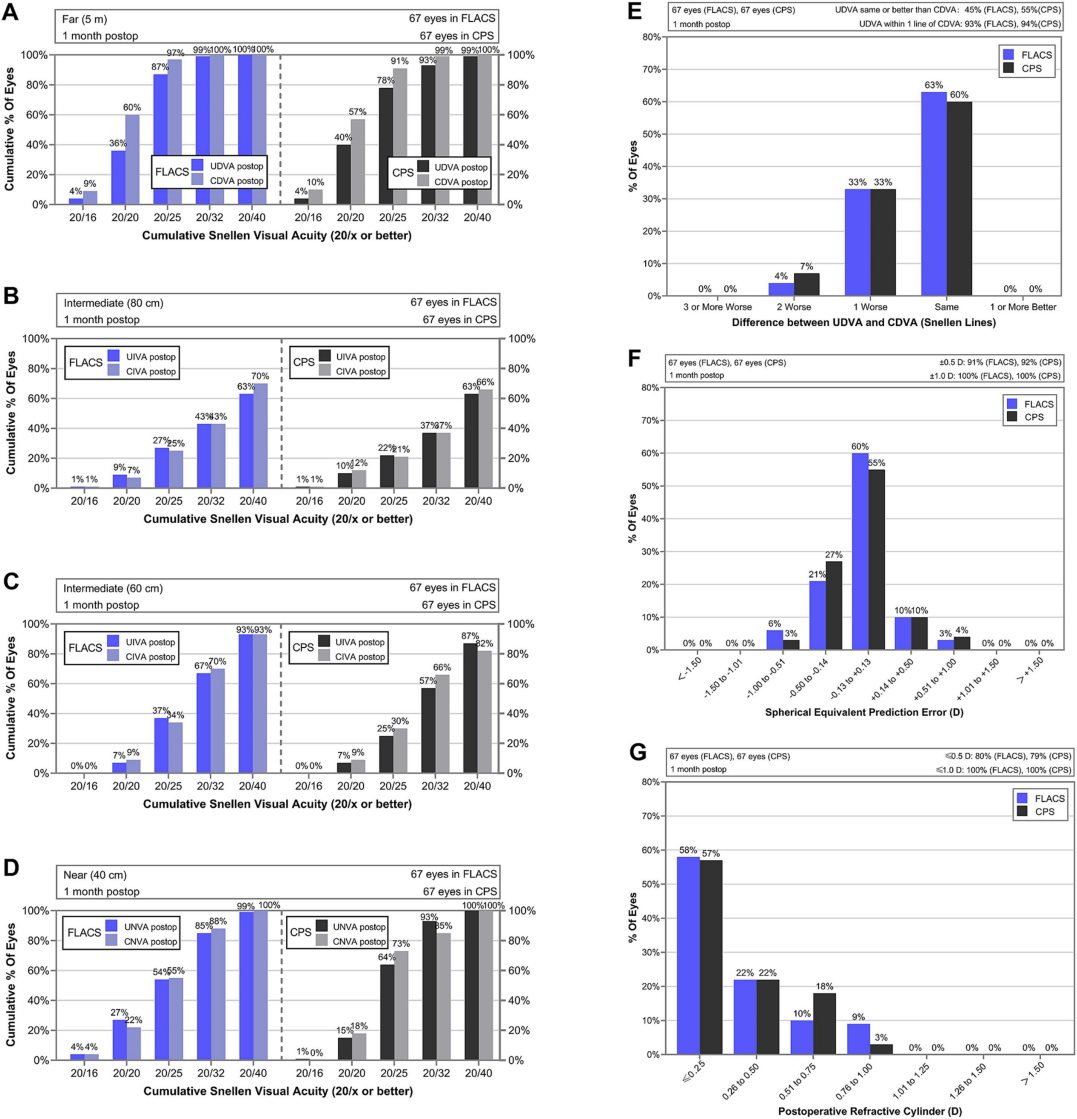
Standard visual outcomes of Tecnis Synergy ZFR00V implantation in FLACS and CPS groups. **(A)** Cumulative percentage of eyes achieving monocular CDVA and UDVA at far distance. **(B)** Cumulative percentage of eyes achieving monocular UIVA and DCIVA at intermediate distance (80 cm). **(C)** Cumulative percentage of eyes achieving monocular UIVA and DCIVA at intermediate distance (60 cm). **(D)** Cumulative percentage of eyes achieving monocular UNVA and DCNVA at intermediate distance (40 cm). **(E)** Difference on Snellen lines in far distance between postoperative UDVA and CDVA. **(F)** Distribution of spherical equivalent prediction error. **(G)** Distribution of postoperative refractive cylinder.

The mean UDVA in the FLACS group was approximately one line better than in the CPS group 1 day postoperatively (0.26 vs. 0.33, *p* = 0.049). At 1 month, no statistically significant differences were observed between the two groups in UDVA, uncorrected intermediate visual acuity (UIVA), and uncorrected near visual acuity (UNVA), nor in corrected distance visual acuity (CDVA), distance-corrected intermediate visual acuity (DCIVA) and distance-corrected near visual acuity (DCNVA) (all *p* > 0.05, Table 3). Cumulative proportions of eyes achieving VA of at least 20/16, 20/20, 20/25, 20/32, and 20/40 at different distance are presented in Figures 1A–D. In both groups, over 90% of eyes exhibited a UDVA within one line of the CDVA (Figure 1E).

**Table 3:** Postoperative refraction and visual outcomes at 1 month.

Parameter	FLACS (*n* = 67)	CPS (*n* = 67)	*p*
	Mean ± SD	Mean ± SD	
SE (D)	−0.07 ± 0.32	−0.06 ± 0.32	0.827^a^
UDVA (5 m)	0.07 ± 0.08	0.09 ± 0.11	0.613^a^
UIVA (80 cm)	0.26 ± 0.15	0.28 ± 0.16	0.566^a^
UIVA (60 cm)	0.20 ± 0.11	0.23 ± 0.13	0.117^a^
UNVA (40 cm)	0.13 ± 0.11	0.13 ± 0.08	0.757^a^
CDVA (5 m)	0.04 ± 0.07	0.04 ± 0.08	0.818^a^
DCIVA (80 cm)	0.26 ± 0.14	0.27 ± 0.15	0.509^a^
DCIVA (60 cm)	0.19 ± 0.11	0.22 ± 0.13	0.369^a^
DCNVA (40 cm)	0.13 ± 0.10	0.12 ± 0.09	0.450^a^

FLACS, femtosecond laser-assisted cataract surgery; CPS, conventional phacoemulsification surgery; SD, standard deviation; SE, spherical equivalent; UDVA, uncorrected distance visual acuity; logMAR, logarithm of the minimum angle of resolution; UIVA, uncorrected intermediate visual acuity; UNVA, uncorrected near visual acuity; CDVA, corrected distance visual acuity; DCIVA, distance-corrected intermediate visual acuity; DCNVA, distance-corrected near visual acuity.

a Wilcoxon–Mann–Whitney U test.

### 3.2 Postoperative refraction

As shown in Table 3, no statistically significant differences in SE were observed between the two groups at 1 month (all *p* > 0.05). The distributions of postoperative SE and residual astigmatism are presented in Figures 1F,G. In both groups, all treated eyes achieved postoperative SE and residual astigmatism within ±1.00 D. The proportion of eyes with postoperative SE within ±0.50 D were 91% in the FLACS group and 92% in the CPS group, respectively, while the proportion with residual astigmatism within ±0.50 D were 80 and 79%, respectively.

### 3.3 Defocus curve

**Figure 2:**
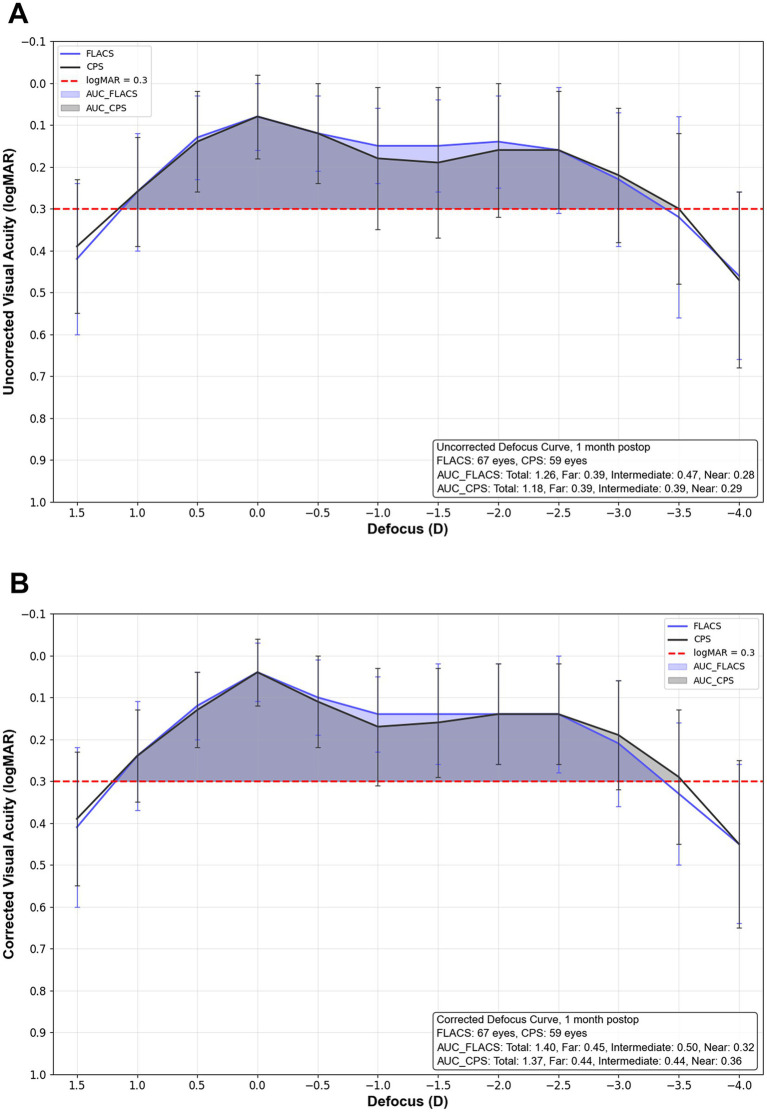
Uncorrected and corrected monocular defocus curve in FLACS and CPS groups. **(A)** Uncorrected defocus curve. **(B)** Corrected defocus curve.

No statistically significant differences in uncorrected or corrected VA were observed at any defocus level from +1.5 D to −4.0 D between the two groups (all *p* > 0.05). As illustrated in Figure 2, the areas under the curve (AUC) below 0.3 logMAR for uncorrected and distance-corrected defocus curves were 1.26 and 1.40 in the FLACS group, compared to 1.18 and 1.37 in the CPS group. Both groups achieved 0.2 logMAR or better VA within a defocus range of +0.5 D to −2.5 D.

### 3.4 Visual quality

**Figure 3:**
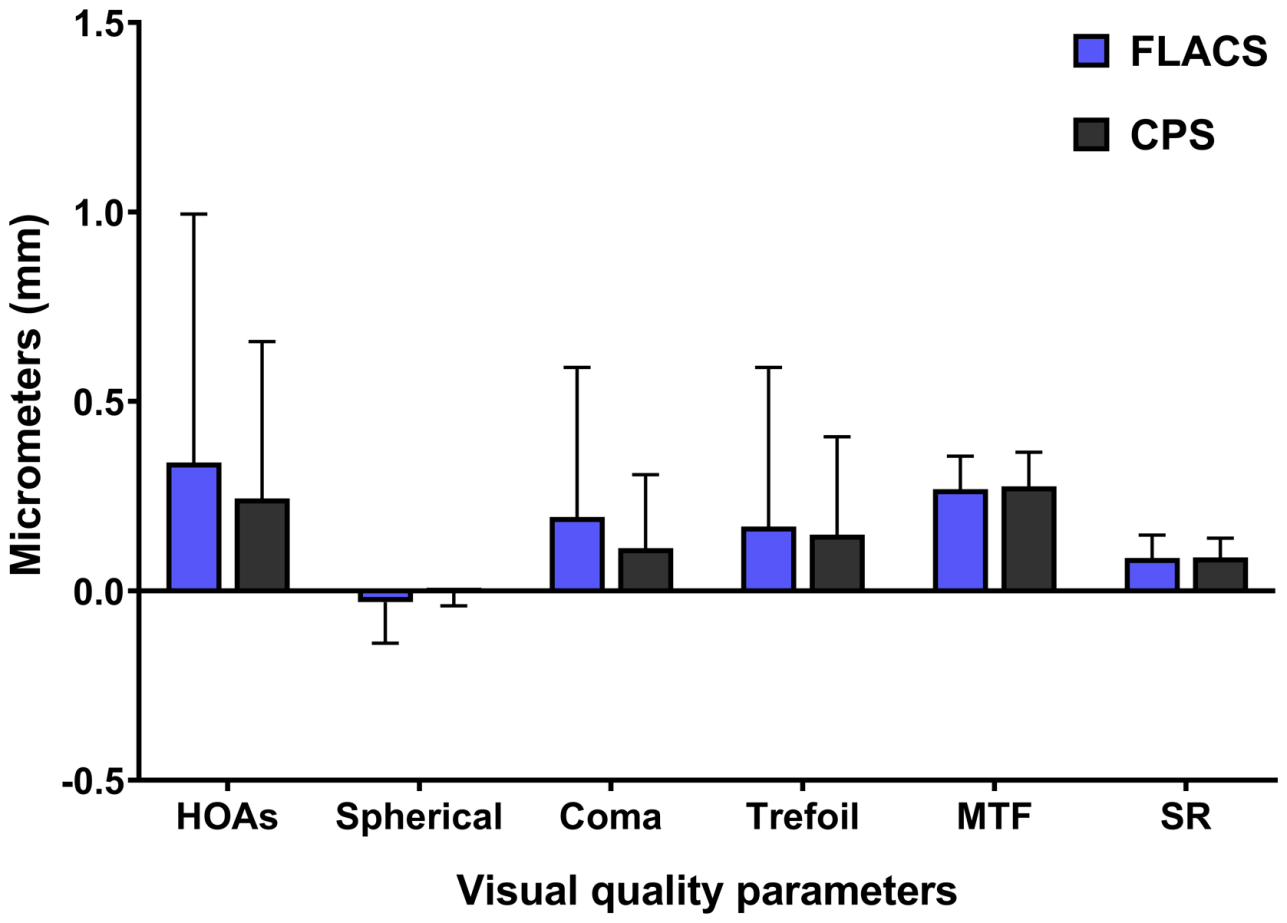
Postoperative ocular aberrations and visual quality parameters at 1 month.

The postoperative total ocular HOAs, MTF, and SR are presented in Table 4. Compared to preoperative values, both groups showed significant improvements in HOAs, coma, trefoil, MTF, and SR at 1 month (all *p* < 0.05). The FLACS group exhibited slightly higher aberrations and marginally lower MTF and SR values than the CPS group (Figure 3). However, no statistically significant differences were detected for any of these visual quality parameters between the two groups (all *p* > 0.05).

**Table 4:** Comparison of postoperative ocular aberrations and visual quality parameters between FLACS and CPS groups.

Parameter	FLACS (*n* = 39)	CPS (*n* = 36)	*p*
	Mean ± SD	Mean ± SD	
HOAs	0.339 ± 0.656^**^	0.244 ± 0.414^**^	0.979^a^
Spherical aberration	−0.030 ± 0.108^**^	−0.005 ± 0.034	0.393^a^
Coma	0.195 ± 0.395^**^	0.113 ± 0.194^**^	0.429^a^
Trifoil	0.170 ± 0.420^**^	0.148 ± 0.259^*^	0.248^a^
MTF	0.268 ± 0.088^**^	0.276 ± 0.090^**^	0.675^a^
SR	0.08664 ± 0.06085^**^	0.08785 ± 0.05131^**^	0.633^a^

FLACS, femtosecond laser-assisted cataract surgery; CPS, conventional phacoemulsification surgery; SD, standard deviation; HOA, higher-order aberrations; MTF, modulation transfer function; SR, Strehl ratio.

a Wilcoxon–Mann–Whitney U test.

^*^p < 0.05, compared to preoperative values, Wilcoxon signed-rank test.

^**^p < 0.01, compared to preoperative values, Wilcoxon signed-rank test.

## 4 Discussion

Cataract surgery has entered the era of refractive surgery. This rapid evolution stems partly from the iterative development of intraocular lenses in recent years. Advances in surgical techniques, including femtosecond laser-assisted cataract surgery, have also been instrumental. However, whether FLACS provides superior postoperative visual acuity and quality of vision compared to CPS remains a subject of debate, especially for patients implanted with presbyopia-correcting IOLs.

In our study, FLACS demonstrated superior full-range and intermediate vision (Figures 1, 2). For uncorrected DC, the AUCs were 7% and 21% higher in the FLACS group the total and intermediate (−0.5 D to −2.0 D) ranges, and a similar pattern was also observed in the distance-corrected DC. A greater proportion of patients in the FLACS group also achieved 20/32 or better VA at 80 cm and 60 cm. While the CPS group showed an 11% higher AUC in the near range (−2.0 D to −4.0 D) for distance-corrected defocus, VA was comparable between groups at the designed reading distance, equivalent to −2.0 D to −3.0 D. Both groups achieved similar VAs at far distance (5 m) and within the far defocus range (+0.5 D to −0.5 D). Overall, the FLACS group provided relatively better and more stable functional intermediate and near vision. The mean corrected visual acuities for the FLACS and CPS groups within the +0.5 D to −2.5 D defocus range were 0.12 and 0.13, respectively. Shin et al. reported that the ZFR00V IOL provided a mean visual acuity of 0.11 or better across this same defocus range, further supporting its ability to deliver satisfactory continuous vision (25).

**Figure 4:**
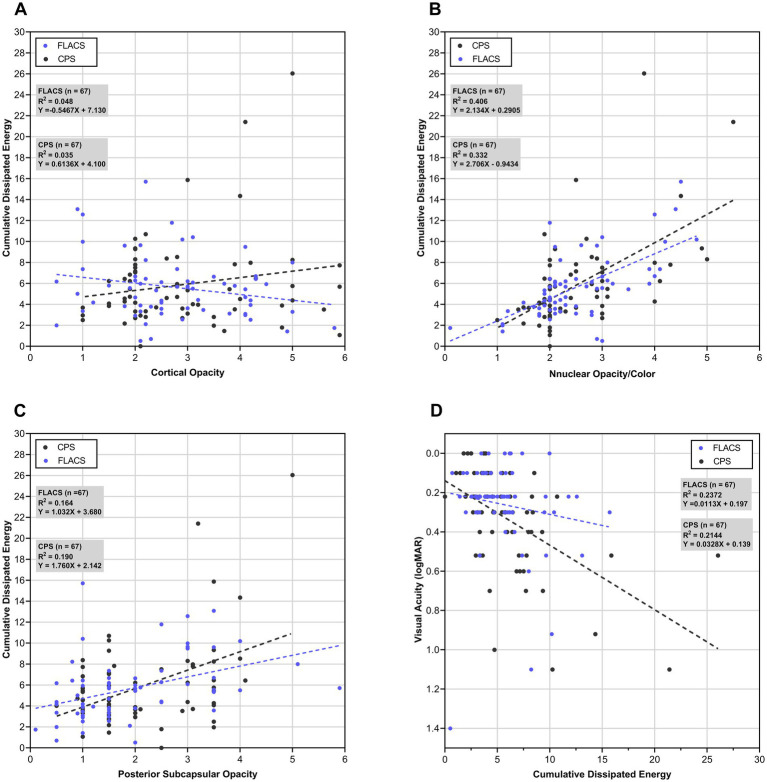
Linear regression analysis of cataract grading, postoperative visual acuity and cumulative dissipated energy (CDE). **(A)** Correlation between cortical opacity and CDE. **(B)** Correlation between nuclear opacity/color and CDE. **(C)** Correlation between posterior subcapsular opacity and CDE. **(D)** Correlation between CDE and postoperative visual acuity at 1 day.

Emulsifying a hard nucleus typically requires higher energy. Linear regression analysis confirmed a significant correlation between nuclear scores and CDE (*p* < 0.001), whereas cortical and posterior subcapsular opacities showed weaker associations (Figures 4A–C). In FLACS, laser-assisted lens fragmentation may reduce the required phacoemulsification energy and effective phaco time, thereby potentially minimizing thermal damage to ocular tissues (7, 26–31). FLACS has demonstrated potential advantages in visual outcomes over CPS primarily in cases of hard nuclear cataract (11, 32). The trend lines in our study also indicated that the CDE was less in the FLACS group when the nuclei were moderate to hard (Figure 4B). However, a contralateral study comparing the two techniques found no significant difference in cumulative dissipated energy for cataracts with a grading score of ≤ 3 (32). In our study, the baseline nuclear grading scores were comparable in FLACS and CPS groups (both 2.5), indicating a relatively soft nuclear profile. Correlation analysis further demonstrated minimal intergroup difference in CDE around a nuclear score of 2.5. Consequently, no significant differences were observed in distance, intermediate, or near visual acuity between the two groups (Table 3). One month postoperatively, the UDVA of FLACS and CPS groups was 0.07 ± 0.08 and 0.09 ± 0.11, respectively, which was largely consistent with previous studies (22, 25, 33–35). Shin et al. reported a UDVA of 0.06 ± 0.07 at 3 months postoperatively with the ZFR00 IOL (25), while Moshirfar et al. (33) observed a UDVA of 0.12 ± 0.07 in 69 patients. In our study, both groups exhibited better intermediate VA at 60 cm than at 80 cm, which could be attributed to the intermediate focal length of the ZFR00V IOL being closer to 60 cm (36).

Previous studies have reported less corneal edema and lower corneal endothelial cell loss in FLACS patients during the early postoperative period compared to conventional cataract surgery, which may accelerate VA recovery (10, 26, 28). We hence further analysis the correlation between CDE and VA at 1 day postoperatively (Figure 4D), finding a significant correlation in the CPS group (*p* < 0.001). With comparable CDE between groups, VA recovered faster in the FLACS group at the very early postoperative stage, an effect potentially attributable to milder corneal edema. This early advantage, however, appears negligible by 1 week. Several meta-analyses have reported no significant difference in uncorrected distance visual acuity (UDVA) at 1 week between FLACS and CPS (27, 28, 37).

Femtosecond laser-assisted capsulorhexis demonstrates high precision and stability, potentially preventing an increase in HOAs from operational errors or non-standardized procedures (38–41). Both groups in this study showed significant improvement in visual quality parameters compared to preoperative status (Table 4). Previous research has largely indicated that FLACS holds significant advantages over CPS in objective visual quality metrics (41, 42). In a study involving 261 eyes, the FLACS group demonstrated superior outcomes in terms of IOL centration, total ocular aberrations, HOAs, coma, spherical aberration, and contrast sensitivity compared to the CPS group (40). For toric IOLs, FLACS may result in lower intraocular coma, which is generally associated with IOL tilting (43). On the contrary, in this study, postoperative HOAs were slightly higher in the FLACS group than in the CPS group (*p* > 0.05). This may be attribute to the HOAs were recorded with a pupil size of 3 mm, whereas previous studies reported HOAs under a 4 to 6 mm pupil diameter. The small sample size in this study also made it incompetent to detect significant difference between FLACS and CPS. Zhong et al. (14) observed that in patients implanted with EDOF IOLs, the MTF and SR values in the FLACS group were higher than those in the CPS group, which was consistent with our results. However, a study by Wang et al. reported no difference in postoperative visual quality between FLACS and CPS (44), suggesting that FLACS may not confer superior visual quality outcomes in the absence of significant IOL decentration.

This study has several limitations. First, the sample size was relatively small and the follow-up period was short, as visual outcomes were reported only at 1 month postoperatively. A limited sample size increases the risk of a type II error when comparing visual outcomes between groups, while the short-term follow-up diminishes our ability to evaluate potential long-term advantages of FLACS. Second, the assessment lacked subjective visual quality scores or patient-reported outcome measures. We also did not include complex cases, such as mature cataracts or high myopia, where FLACS may offer particular advantages (11–13, 32, 45). Finally, as a non-randomized retrospective study, patient assignment was based on the surgical technique used. Although we analyzed baseline characteristics including nuclear hardness and IOP and found no significant intergroup differences, heterogeneity in other parameters, such as corneal endothelial cell density and systemic conditions, may still have influenced the analysis, thereby compromising the study’s reliability.

In conclusion, patients in both groups achieved favorable continuous vision and improved visual quality following ZFR00V IOL implantation. Compared to CPS, FLACS provided superior full-range vision, along with enhanced and more stable intermediate and near vision. FLACS also demonstrated advantages in the early recovery of visual acuity. However, future randomized, prospective studies with larger samples, longer follow-up, inclusion of complicated cases, and visual quality questionnaires are needed to enable more nuanced comparisons and stratified analyses.

## Data Availability

The original contributions presented in the study are included in the article/supplementary material, further inquiries can be directed to the corresponding author.
